# The incidence and risk factors of selected drug prescriptions and outpatient care after SARS-CoV-2 infection in low-risk subjects: a multicenter population-based cohort study

**DOI:** 10.3389/fpubh.2023.1241401

**Published:** 2023-10-04

**Authors:** Carlo Gagliotti, Federico Banchelli, Angela De Paoli, Rossella Buttazzi, Elena Narne, Enrico Ricchizzi, Elena Schievano, Stefania Bellio, Gisella Pitter, Michele Tonon, Lorenzo Maria Canziani, Maurizia Rolli, Evelina Tacconelli, Elena Berti, Francesca Russo, Maria Luisa Moro

**Affiliations:** ^1^Department of Innovation in Healthcare and Social Services, Emilia-Romagna Region, Bologna, Italy; ^2^Azienda Zero, Padova, Italy; ^3^Directorate of Prevention, Food Safety, and Veterinary Public Health, Venezia, Italy; ^4^Department of Diagnostics and Public Health, University of Verona, Verona, Italy

**Keywords:** SARS-CoV-2, post-COVID, COVID-19 sequelae, outpatient care, drug prescriptions, low-risk subjects, population-based cohort, ORCHESTRA project

## Abstract

**Background:**

Knowledge about the dynamics of transmission of SARS-CoV-2 and the clinical aspects of COVID-19 has steadily increased over time, although evidence of the determinants of disease severity and duration is still limited and mainly focused on older adult and fragile populations.

**Methods:**

The present study was conceived and carried out in the Emilia-Romagna (E-R) and Veneto Regions, Italy, within the context of the EU’s Horizon 2020 research project called ORCHESTRA (Connecting European Cohorts to increase common and effective response to SARS-CoV-2 pandemic) (www.orchestra-cohort.eu). The study has a multicenter retrospective population-based cohort design and aimed to investigate the incidence and risk factors of access to specific healthcare services (outpatient visits and diagnostics, drug prescriptions) during the post-acute phase from day-31 to day-365 after SARS-CoV-2 infection, in a healthy population at low risk of severe acute COVID-19. The study made use of previously recorded large-scale healthcare data available in the administrative databases of the two Italian Regions. The statistical analysis made use of methods for competing risks. Risk factors were assessed separately in the two Regions and results were pooled using random effects meta-analysis.

**Results:**

There were 35,128 subjects in E-R and 88,881 in Veneto who were included in the data analysis. The outcome (access to selected health services) occurred in a high percentage of subjects in the post-acute phase (25% in E-R and 21% in Veneto). Outpatient care was observed more frequently than drug prescriptions (18% vs. 12% in E-R and 15% vs. 10% in Veneto). Risk factors associated with the outcome were female sex, age greater than 40 years, baseline risk of hospitalization and death, moderate to severe acute COVID-19, and acute extrapulmonary complications.

**Conclusion:**

The outcome of interest may be considered as a proxy for long-term effects of COVID-19 needing clinical attention. Our data suggest that this outcome occurs in a substantial percentage of cases, even among a previously healthy population with low or mild severity of acute COVID-19. The study results provide useful insights into planning COVID-19-related services.

## 1. Introduction

The COVID-19 pandemic has caused a global health, social and economic emergency (1). Over time, knowledge about the dynamics of transmission of SARS-CoV-2 and the clinical aspects of COVID-19 has steadily increased, although evidence of the determinants of disease severity and duration is still limited and mainly focused on older adult and fragile populations. In vulnerable populations there is also an increasing number of reports showing that individuals with comorbidities or admitted to the ICU for severe SARS-CoV-2 infection have a higher incidence of long-term effects of COVID-19 (2–6). Symptoms of sequelae vary widely in type and timing, as they can follow initial recovery or persist from the acute episode. They may also fluctuate, relapse, or change over time (6–9). The National Institute for Health and Care Excellence (NICE) provided clinical case definitions to identify and diagnose the long-term effects of COVID-19. Ongoing symptomatic COVID-19 is defined as the presence of persistent COVID-19 signs and symptoms after 4 weeks from diagnosis and up to 12 weeks. Post-COVID-19 syndrome is the presence of signs and symptoms that developed during or after a SARS-CoV-2 infection and persist for more than 12 weeks, not explained by an alternative diagnosis (8). Another definition was provided by the World Health Organization (WHO). According to WHO, the post COVID-19 condition occurs in individuals with a history of probable or confirmed SARS CoV-2 infection, usually 3 months from the onset of COVID-19 with symptoms and that last for at least 2 months and cannot be explained by an alternative diagnosis (9). Effects of ongoing symptomatic and post-COVID-19 include fatigue/weakness, dyspnea, decreased exercise tolerance, cognitive impairment, prolonged smell and taste disorders, headache, anxiety, depression, insomnia, arthromyalgia, diabetes, and renal sequelae (2, 4–8, 10–16). These symptoms tend to cluster and can lead to a lower perceived quality of life and to an increased demand for and access to specific healthcare services (8, 14). The etiology of long-term effects of COVID-19 is likely multifactorial, with endothelial damage and immunological phenomena playing a significant role (7). Female sex, older age, presence of comorbidities, and severity in the acute phase of COVID-19 are associated with an increased risk of ongoing symptomatic and post-COVID-19 syndrome (3–5). Major limitations of previous studies include the study population, sample size, length of follow-up, case definition, and study design (3, 17). A few reports have recently highlighted diagnoses of long COVID in outpatients and/or previously healthy populations (18, 19). The present study was conceived and carried out in the Emilia-Romagna (E-R) and Veneto Regions, Italy, within the context of the EU’s Horizon 2020 research project called ORCHESTRA (Connecting European Cohorts to increase common and effective response to SARS-CoV-2 pandemic).^1^ The major aim of the study is to assess the incidence and risk factors of access to specific healthcare services (outpatient visits and diagnostics, drug prescriptions) within 12 months from confirmed SARS-CoV-2 diagnosis in a healthy population at low risk of severe acute COVID-19.

## 2. Materials and methods

### 2.1. Data source

The study was carried out in E-R and Veneto, two neighboring regions of northern Italy with a population of approximately 4.4 and 4.9 million residents, respectively. Both these two regions oversee a regional healthcare system and have exclusive competence in regulating, financing, and organizing healthcare services and activities carried out within their territory. Data were extracted from the E-R and Veneto Regions healthcare administrative databases. They include comprehensive information about healthcare provision by the regional healthcare systems. Secure record-linkage procedures were carried out at the individual level to merge pseudonymized data related to: official notifications of SARS-CoV-2 infections; drug prescriptions; outpatient care; residence and vital status; acute hospital admissions; community hospital admissions; emergency room access; long-term care facilities; and integrated home care. In the E-R cohort, an individual risk of hospitalization and death score was also assigned using a previously developed standardized algorithm. This algorithm relies on a multivariable prediction model which estimates a punctual measure for the individual risk of hospitalization or death within the reference year. The risk of hospitalization and death is then scored according to a four-level scale: low (probability <6%), moderate (≥ 6% and < 15%), high (≥ 15% and < 25%), very high (≥ 25%). This score is assigned yearly based on demographic and residence characteristics, comorbidities, and access to a wide spectrum of healthcare resources in a multiyear period before the reference year, and is routinely available in E-R administrative databases (20). The extracted data include updates of databases up to November 2021 for E-R and up to December 2021 for Veneto.

### 2.2. Study Aim and design

The study is a multicenter retrospective population-based cohort study aiming to investigate the incidence and risk factors of access to healthcare services. It focused on a largely healthy population, at low risk of acute severe COVID-19, to ensure that a high fraction of outcomes is attributable to COVID-19. Eligible participants included all adult subjects aged ≥18 years at diagnosis and with continuous residence status in the two regions in the 365 days before diagnosis. Low risk of severe acute COVID-19 was characterized by the absence of all the following types of care, in the 365 days prior to the SARS-CoV-2 diagnosis: hospitalization; visits to the emergency room; integrated home care; residence in a long-term care facility; selected drug prescriptions (at least one drug within the selected list; see Supplementary Table 1); selected outpatient care (at least one visit/diagnostics within the selected list; see Supplementary Table 1). Additionally, in the E-R cohort, low risk of severe acute COVID-19 was assigned only to subjects with a low-to-moderate risk of hospitalization and death score (19). The population of interest included all consecutive adult individuals with confirmed SARS-CoV-2 infection (PCR or antigen tests) in the E-R and Veneto Regions between February 2020 and November 2020 (E-R) or December 2020 (Veneto) who, at the time of diagnosis, were at low risk of severe acute COVID-19 disease. None of these subjects was vaccinated at the time of SARS-CoV-2 diagnosis, as the vaccination campaign in Italy only started in late December 2020. Individuals entered the cohort on the day of SARS-CoV-2 diagnosis. Outcomes were assessed within 365 days before and after the diagnosis. In the follow-up period we distinguished an acute phase (AP) from diagnosis till day 30 and a post-acute phase (PAP) from day 31 till day 365. For patients who were hospitalized at day 30, PAP started on the first day after hospital discharge. Outcomes were assessed during the PAP. Individuals who were continuously hospitalized from the AP to more than 365 days after SARS-CoV-2 diagnosis, or who had moved their residence outside the region during the AP, were excluded. Duplicate or incomplete records were discarded. The study was carried out and reported according to the GATHER statement (21).

### 2.3. Study outcomes

The outcome of interest was the access to specific healthcare services during the PAP, defined through a selected list of drug prescriptions and outpatient visits and diagnostics. This combined outcome was considered a proxy for the long-term effects of COVID-19 requiring medical attention (22). A broad range of drug prescriptions and outpatient care services was considered, to reflect the multifactorial nature of COVID-19, which can affect several organs and systems. Selected drug prescriptions included cardiovascular system (e.g., antithrombotics, antiarrhythmics, antihypertensives, beta blockers), antidiabetic, nervous system (e.g., antidepressants), and respiratory system (e.g., adrenergics and other drugs for obstructive airway diseases) drugs, oxygen and corticosteroids (22–24). Selected outpatient visits and diagnostics included ambulatory visits in cardiology, pneumology, angiology, neurology, psychiatry, rehabilitation-motor, nephrology, and diabetes, as well as other diagnostic and therapeutic procedures such as chest imaging, cardiac ultrasound imaging, pneumological diagnostics, electrocardiography, oxygen therapy, respiratory, and cardiological rehabilitation, peripheral vascular ultrasound imaging, training for cognitive disorders, hemodialysis, renal imaging, and glycated hemoglobin analysis (22–24). Only drugs and outpatient care provided by the regional healthcare system were included in the analysis. Drugs that are not reimbursed as well as private outpatient care not provided by the regional healthcare systems are not recorded. Details of drugs and outpatient care are reported in Supplementary Table 1, together with extraction criteria based on ATC classification and on E-R regional outpatient codes. Two analyses were performed, each with a different definition of the outcome variable. In the first analysis we considered a combined outcome including selected drug prescriptions and selected outpatient care, whichever came first. In the second analysis, each of the two outcomes of interest (drug prescriptions and outpatient care) was analyzed separately and the other was considered neither as a competing event nor as censoring. Hospitalization and death were always considered as competing events following a competing risk analysis framework. Hospitalization was considered a competing event due to drug prescriptions and clinical consulting not being tracked at the individual level during acute care stay: This prevented us from observing these events in administrative databases during the period a patient was hospitalized.

### 2.4. Other extraction criteria

**Figure 1:**
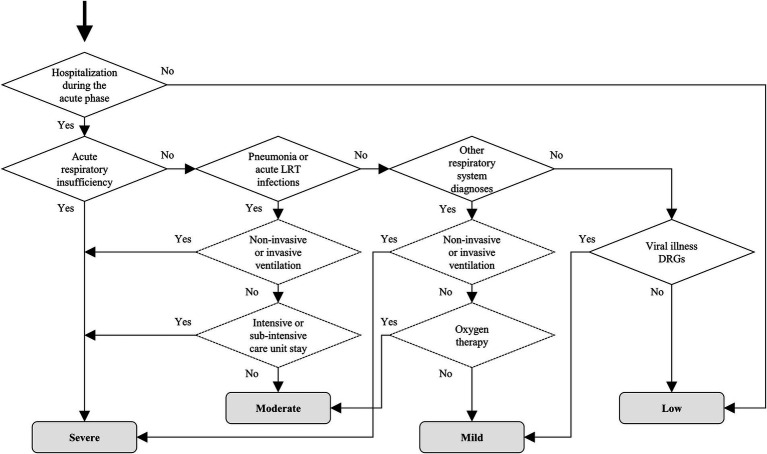
Algorithm for the assignment of COVID-19 severity. Notes: The decision tree shows the algorithm for the assignment of COVID-19 severity during the acute phase. The rhombuses indicate binary decision rules based on hospitalizations, respiratory diagnoses, Diagnosis-related groups (continuous border line) and ventilation procedures (dotted border line). The gray boxes indicate the assigned COVID-19 severity level (low, mild, moderate, severe). The data extraction criteria for each element in the decision tree are reported in Supplementary Table 2. LRT = lower respiratory tract; DRG = Diagnosis-related group.

Some clinically relevant variables were determined based on data recorded in administrative databases. COVID-19 severity (expressed on a four-level ordinal scale as low, mild, moderate or severe) was algorithmically assigned. The algorithm was based on respiratory system diagnoses (i.e., acute respiratory insufficiency, pneumonia, acute lower respiratory tract infections, other respiratory diagnoses), on ventilation procedures administered (i.e., oxygen therapy, non-invasive ventilation, invasive ventilation), and on intensive or sub-intensive care unit stay during hospitalizations in the AP. For subjects not hospitalized in the AP, the “low” level of severity was assigned. The algorithm is reported in Figure 1. Acute extrapulmonary complications (i.e., vascular, hemorrhagic, thrombotic, cardiac, neurological, septicemia and acute organ failure) were also identified, based on diagnoses during hospitalizations in the AP. These variables can occur only for hospitalized subjects. Criteria for the identification of respiratory diagnoses, ventilation procedures and acute extrapulmonary Complications, based on ICD-9-CM codes, are reported in Supplementary Table 2.

### 2.5. Statistical analysis

The statistical analysis was carried out separately in the two Regions, and results were pooled using meta-analysis. The frequency distributions of the categorical characteristics were described as absolute and percentage numbers. The numerical variables were described as the mean ± standard deviation and range. To evaluate potential risk factors, multivariable regression models were carried out. The incidence of outcomes in the PAP was analyzed with a competing risk approach: selected drug prescriptions and/or selected outpatient care were the outcomes of interest, whereas hospitalization and death were the competing events. Individuals who moved their residence outside of the E-R region during the PAP, as well as those who did not experience any outcome or competing event by the end of the PAP, were treated as having censored follow-up times. The incidence of outcomes over time was described using cumulative incidence functions curves (25). Uncertainty in curves was expressed with 95% confidence interval (CI) calculated with the asymptotic Aalen method (26). A multivariable Fine-Gray (FG) proportional subdistribution hazard regression model was used to assess the relationship between subjects’ characteristics and the hazard of outcomes over time, also accounting for the occurrence of competing events (27). The explanatory variables were: time period (1st wave: February–May 2020, intermediate period: June–September 2020, 2nd wave: October–December 2020); age class at diagnosis (18–39, 40–49, 50–59, 60–69, 70–79, ≥80 years); sex (male, female); Italian citizenship (yes, no); only in the E-R cohort, the risk of hospitalization and death score (low, moderate); COVID-19 severity during the AP (low, mild, moderate, severe); acute extrapulmonary complications occurring in hospital during the AP (yes, no). Moreover, the area of residence (8 Local Health Units in E-R and 9 in Veneto) was also used as an independent variable to account for potentially different health policies. Associations were measured using the subdistribution hazard ratio (HR) and the uncertainty in results was expressed with 95% CI. Cis for HRs were calculated with the Wald method based on normal approximation. Pooling of hazard ratios obtained in the E-R and Veneto Regions’ cohorts was carried out using random effects meta-analysis (MA). MA was performed with the inverse variance weights method and maximum likelihood estimator for between-study variance. Between-cohort heterogeneity was measured with the tau statistic (28) and its significance was assessed with the Cochran’s Q test. All statistical tests were two-sided. Analyses were carried out by E-R with SAS/STAT 15.1 (SAS Institute Inc., Cary, NC) and R 4.0.4 (The R Foundation for Statistical Computing, Wien) and by Veneto with SAS/STAT 13.1 statistics software.

## 3. Results

**Figure 2:**
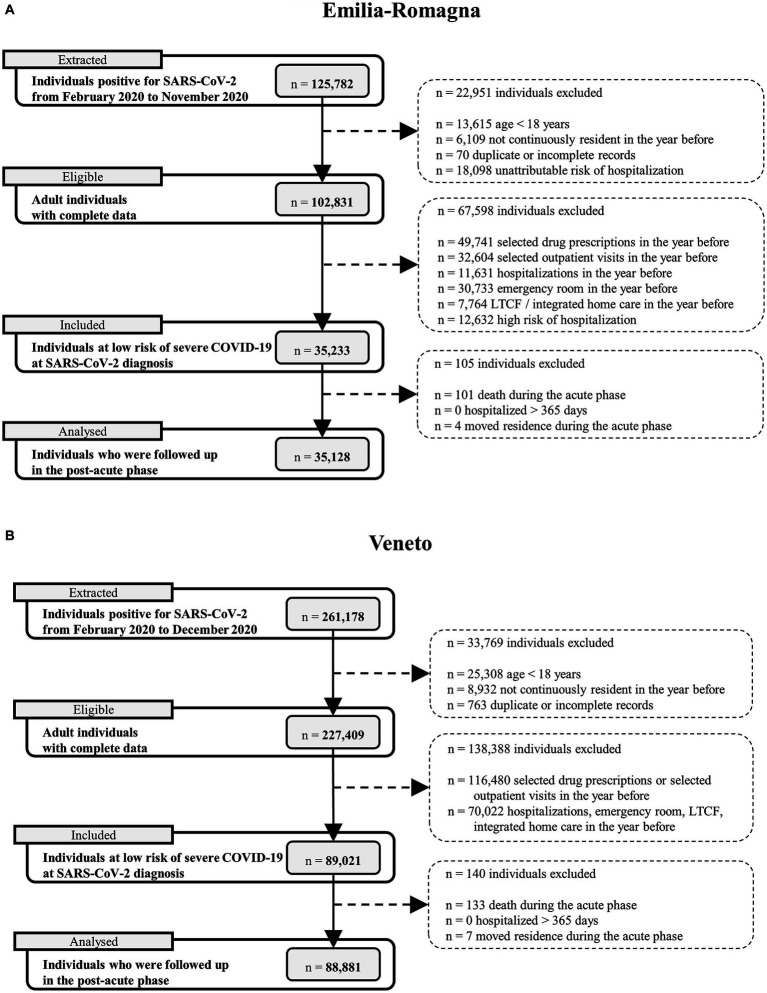
Flow charts describing selection of subjects in the two cohorts. Notes: **(A)** = Emilia-Romagna Region; **(B)** = Veneto Region. The total number of excluded individuals is the number of subjects who had at least one of the specific exclusion criteria listed in the flow-chart.

There were 125,782 individuals positive for SARS-CoV-2 in the E-R region in the period from February 2020 to November 2020, and 261,178 in the Veneto Region from February 2020 to December 2020. Of these, 81.8 and 87.1% were adult individuals with complete data. Those who fulfilled the inclusion and exclusion criteria for the low-risk cohort and who were included in the data analysis numbered 35,128 in Emilia-Romagna and 88,881 in Veneto (Figure 2).

### 3.1. Characteristics of individuals

Descriptive characteristics of the cohort are reported in Table 1. Most subjects were diagnosed for SARS-CoV-2 in Emilia-Romagna in October–November 2020 (80.3%) and in Veneto in October–December 2020 (91.8%), during the second epidemic wave in Italy. The average age at diagnosis was 41.1 ± 14.1 years (range: 18–91) in Emilia-Romagna and 41.8 ± 14.1 years (range: 18–97). Those aged more than 60 years were a small part (9.1 and 10.0%, respectively). About half of the included individuals were female (50.3 and 49.9%) and the most part had Italian citizenship (88.7 and 89.6%). At baseline, the risk of hospitalization and death score estimated in E-R was low for 98.7% and moderate for 1.3% of subjects. During the AP, 96.1% of low-risk individuals in Emilia-Romagna and 98.7% in Veneto experienced low severity COVID-19. Conversely, 0.2% and less than 0.1% of subjects had mild severity, 1.4 and 0.6% had moderate severity, and 2.2 and 0.7% experienced severe COVID-19 disease. Those who were hospitalized during the AP (in acute care hospitals or community hospitals) were 4.3 and 1.6%. Acute extrapulmonary complications have occurred in 0.3 and 0.2% of individuals (Table 1). The higher frequency of individuals who were not hospitalized and who were assigned the lowest severity level in the Veneto cohort likely reflects the wider use that was made of diagnostic testing in 2020 in that Region to detect positive cases even among asymptomatic people (29).

**Table 1:** Characteristics of subjects at low risk of severe COVID-19 disease.

		Emilia-Romagna (*N* = 35,128)		Veneto (*N* = 88,881)	
		n	%	n	%
*Baseline characteristics*					
SARS-CoV-2 diagnosis period	1st wave	4,527	12.9%	4,010	4.0%
	Intermediate	2,384	6.8%	3,305	3.7%
	2nd wave	28,217	80.3%	81,566	91.8%
Sex	Female	17,662	50.3%	44,356	49.9%
Age at diagnosis	18–39	15,943	45.4%	38,795	43.7%
	40–49	8,797	25.0%	21,808	24.5%
	50–59	7,197	20.5%	19,396	21.8%
	60–69	2,370	6.7%	6,678	7.5%
	70–79	674	1.9%	1,742	2.0%
	80–91	147	0.4%	462	0.5%
Italian citizenship	Yes	31,154	88.7%	79,601	89.6%
Risk of hospitalization and death score	Low	34,670	98.7%	-	-
	Moderate	458	1.3%	-	-
*Acute phase characteristics* ^a^					
COVID-19 severity	Low	33,765	96.1%	87,703	98.7%
	Mild	84	0.2%	38	0.0%
	Moderate	498	1.4%	526	0.6%
	Severe	781	2.2%	614	0.7%
Oxygen therapy	Yes	711	2.0%	701	0.8%
Non-invasive ventilation	Yes	139	0.4%	180	0.2%
Invasive ventilation	Yes	103	0.3%	88	0.1%
Intensive care unit stay	Yes	161	0.5%	137	0.2%
Sub-intensive care unit stay	Yes	52	0.1%	0	0.0%
Pneumonia or acute LRT infections	Yes	1,267	3.6%	1,134	1.3%
Acute respiratory insufficiency	Yes	756	2.2%	603	0.7%
Other respiratory infections	Yes	40	0.1%	12	0.0%
Hospitalization	Yes	1,507	4.3%	1,412	1.6%
Acute extrapulmonary complications^b^	Yes	112	0.3%	149	0.2%
Vascular complication	Yes	11	0.0%	92	0.1%
Cardiac complication	Yes	18	0.1%	9	0.0%
Neurological complication	Yes	1	0.0%	4	0.0%
Septicemia	Yes	37	0.1%	36	0.0%
Acute organ failure complication	Yes	55	0.2%	19	0.0%

Notes: LRT = lower respiratory tract; ^a^ = the following variables can only occur for hospitalized patients; ^b^ = acute extrapulmonary complications include the five types of complications which are listed.

### 3.2. Incidence of drug prescriptions and outpatient care

**Figure 3:**
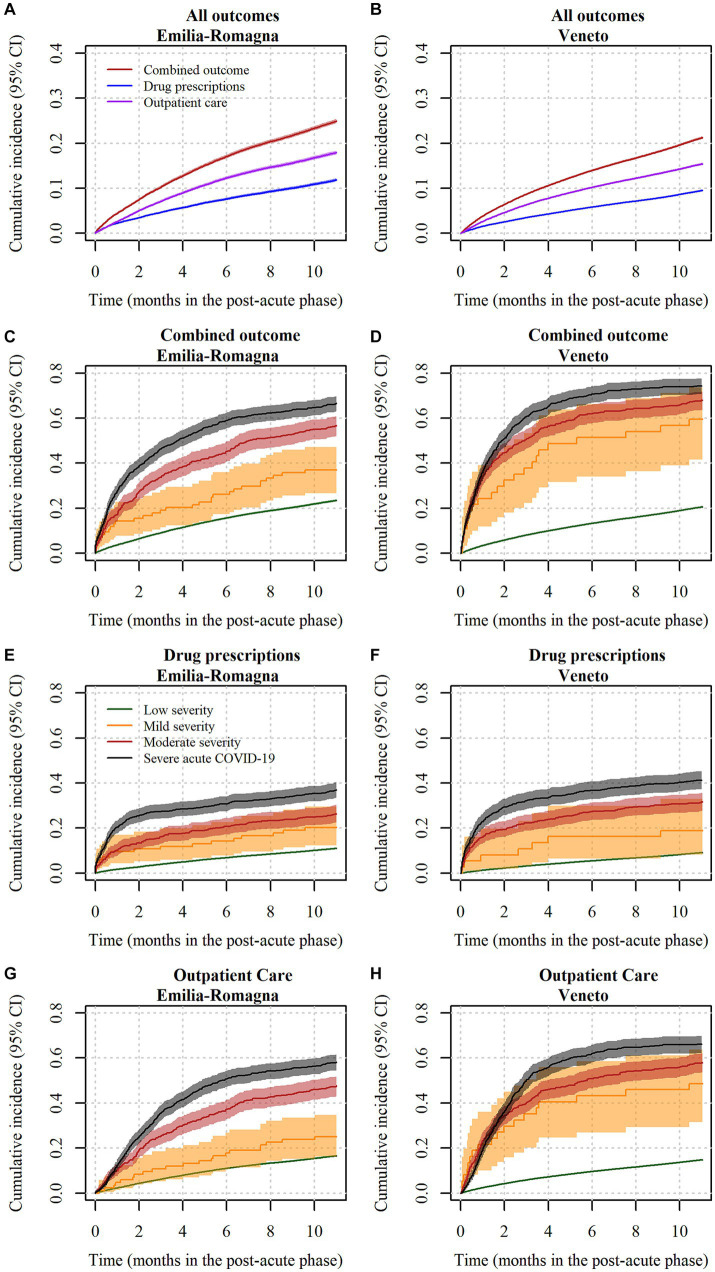
Cumulative incidence curves of drug prescriptions and outpatient care in subjects at low risk of severe COVID-19 disease, by COVID-19 severity. Notes: **(A)** = incidence of outcomes in Emilia-Romagna; **(B)** = incidence of outcomes in Veneto; **(C)** = incidence of the combined outcome in Emilia-Romagna, by COVID-19 severity; **(D)** = incidence of the combined outcome in Veneto, by COVID-19 severity; **(E)** = incidence of selected drug prescriptions in Emilia-Romagna, by COVID-19 severity; **(F)** = incidence of selected drug prescriptions in Veneto, by COVID-19 severity; **(G)** = incidence of selected outpatient care in Emilia-Romagna, by COVID-19 severity; **(H)** = incidence of selected outpatient care in Veneto, by COVID-19 severity. In sub-figures **(A)** and **(B)**, red lines indicate the composite outcome, blue lines indicate the drug prescription outcome, and purple lines indicate the outpatient care outcome. In sub-figures from **(C–H)**, green lines indicate low severity subjects, yellow lines indicate mild severity subjects, red lines indicate moderate severity subjects and black lines indicate severe subjects. In all sub-figures, lines indicate punctual estimates of the cumulative incidence function and areas represent 95% confidence intervals. Confidence intervals were calculated with the asymptotic Aalen method. CI = confidence interval.

The total follow-up time in the PAP was equal to 26,826.7 person-years in E-R and 68,333.1 in Veneto. During this time, 9,208 (26.2%) of low-risk individuals in E-R and 19,769 (22.2%) in Veneto experienced at least one outcome of interest during the PAP. 4,633 (13.2%) and 9,409 (10.6%) subjects, respectively, had at least one selected drug prescription. On the other hand, 6,523 (18.6%) and 14,108 (15.9%) individuals had at least one selected outpatient care visit or diagnostic procedure, respectively. In the competing risks analysis, as reported in Figure 3, the cumulative incidence of the combined outcome at 11 months in the PAP was equal to 24.9% (95% CI = 24.5–25.4%) in E-R and to 21.2% (95% CI = 20.9–21.5%) in Veneto. Drug prescriptions were less frequent than outpatient care: at 11 months in the PAP, the incidence of the former was 11.9% (95% CI = 11.5–12.2%) in E-R and 9.5% (95% CI = 9.3–9.7%) in Veneto, whereas the incidence of the latter was 17.9% (95% CI = 17.5–18.3%) and 15.4% (95% CI = 15.1–15.6%), respectively. Monthly outcomes incidence data are reported in Supplementary Table 3. Competing events such as hospitalization and mortality occurred during the PAP in 1,292 (3.7%) and 26 (0.1%) individuals in E-R, and in 2,837 (3.2%) and 80 (0.1%) individuals in Veneto. The frequency of individuals who accessed healthcare services during the PAP is reported in detail in Table 2. Among selected drug prescriptions, cardiovascular system drugs (6.0% in E-R and 4.7% in Veneto), followed by corticosteroids for systemic use (4.8 and 3.9%) and respiratory system drugs (2.3 and 1.8%) were administered more frequently during the PAP. Among selected outpatient ambulatory services, the most frequent during the PAP were cardio-respiratory visits and procedures (11.6% in E-R and 9.1% in Veneto), followed by diabetic ambulatory visits or procedures (3.9 and 4.3%), and rehabilitation-motor visits (2.0 and 2.3%).

**Table 2:** Access to healthcare services of subjects at low risk of severe COVID-19 disease during the post-acute phase.

Type of care	Emilia-Romagna (*N* = 35,128)		Veneto (*N* = 88,881)	
	n	%	n	%
Combined outcome	9,208	26.2%	19,769	22.2%
Selected drug prescriptions	4,633	13.2%	9,409	10.6%
Selected outpatient care	6,523	18.6%	14.108	15.9%
Drug prescriptions	15,513	44.2%	33,622	37.8%
Cardiovascular system / antithrombotic^a^	2,114	6.0%	4,202	4.7%
Antidiabetic^a^	165	0.5%	256	0.3%
Nervous system^a^	563	1.6%	1,036	1.2%
Respiratory system^a^	798	2.3%	1,580	1.8%
Oxygen^a^	6	0.0%	32	0.0%
Corticosteroids^a^	1,671	4.8%	3,433	3.9%
Antibacterial	5,936	16.9%	13,219	14.9%
Hydroxychloroquine	48	0.1%	131	0.2%
Other drugs	11,208	31.9%	22,699	25.5%
Outpatient care	23,248	66.2%	36,965	41.6%
Cardio-respiratory^b^	4,059	11.6%	8,103	9.1%
Vascular^b^	949	2.7%	1,515	1.7%
Neuro-psychiatric^b^	668	1.9%	1,271	1.4%
Rehabilitation-motor^b^	685	2.0%	2,005	2.3%
Nephrology^b^	33	0.1%	58	0.1%
Diabetes^b^	1,362	3.9%	3,805	4.3%
Other visits / procedures	22,590	64.3%	32,214	36.2%
Acute care hospitalization	1,292	3.7%	2,837	3.2%
Community hospital	3	0.0%	4	0.0%
Long-term care facility	10	0.0%	49	0.1%
Emergency room	5,099	14.5%	12,738	14.3%
Integrated home care	54	0.2%	350	0.4%

Notes: ^a^ = included in selected drug prescriptions outcome; ^b^ = included in selected outpatient care outcome. The sum of individual items may differ from the totals, as subjects may have more than one outpatient care episode or drug prescription during the post-acute phase.

### 3.3. Assessment of risk factors for drug prescriptions and outpatient care

**Figure 4:**
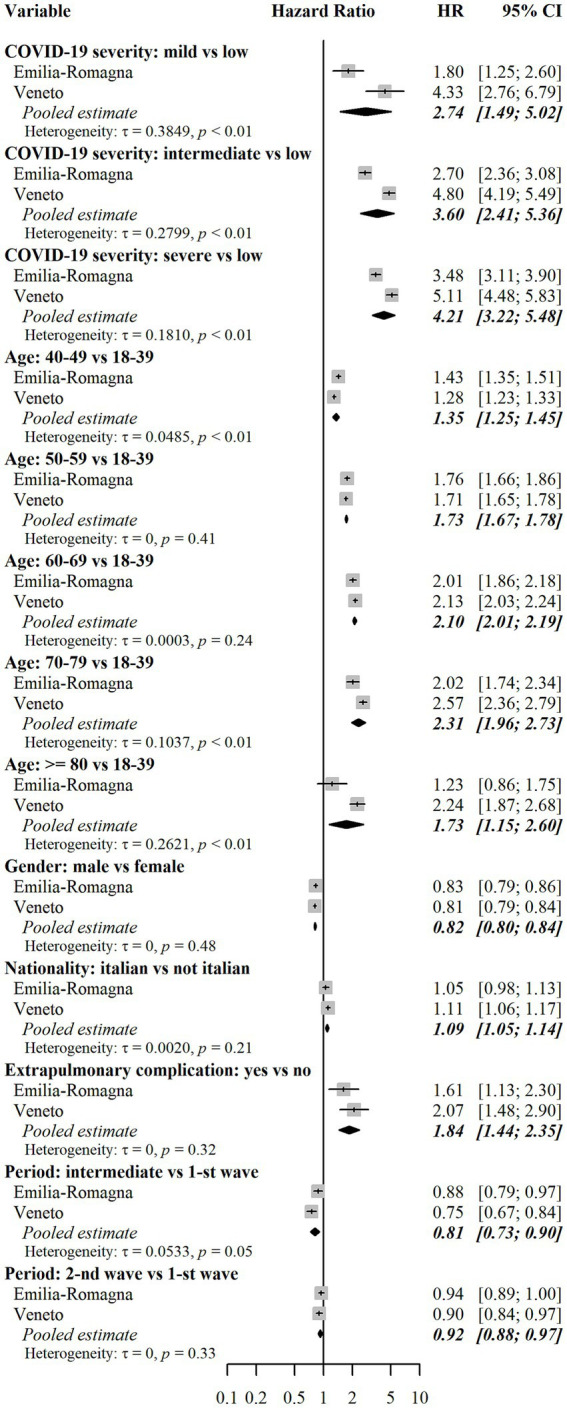
Assessment of risk factors for the combined outcome. Notes: Results for each cohort were estimated using multivariable Fine-Gray subdistribution hazard models. Confidence intervals for hazard ratios in the two cohorts were calculated with the Wald method based on normal approximation and were two-sided. Pooled results were estimated using random effects meta-analysis with inverse variance weights and maximum likelihood estimator for between-study variance. The models also included the following independent variables: risk of hospitalization and death score (only in the E-R cohort), and Local Health Units (8 in E-R and 9 in Veneto). Additional results for these variables are reported in Supplementary Table 4. The combined outcome includes selected drug prescriptions and selected outpatient care, whichever came first. Heterogeneity was measured with the tau (τ) statistic and its significance was assessed with the Cochran’s *Q* test. HR = subdistribution hazard ratio. CI = confidence interval. *p* = *p*-value.

**Figure 5:**
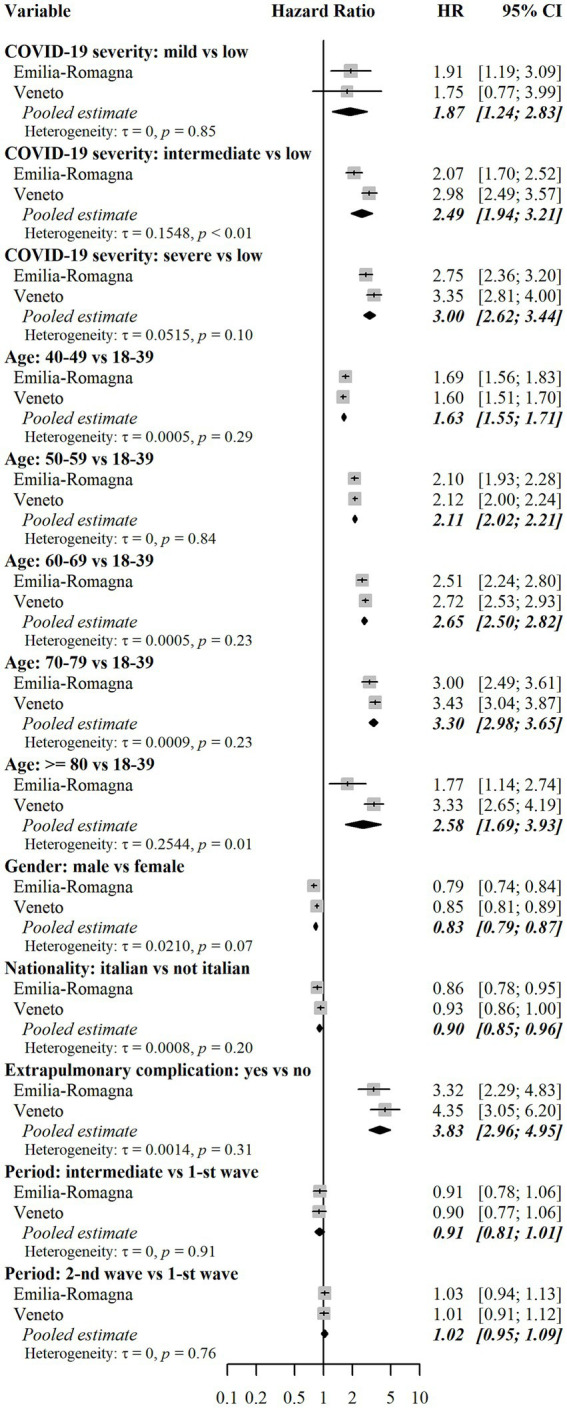
Assessment of risk factors for drug prescriptions. Notes: Results for each cohort were estimated using multivariable Fine-Gray subdistribution hazard models. Confidence intervals for hazard ratios in the two cohorts were calculated with the Wald method based on normal approximation and were two-sided. Pooled results were estimated using random effects meta-analysis with inverse variance weights and maximum likelihood estimator for between-study variance. The models also included the following independent variables: risk of hospitalization and death score (only in the E-R cohort), and Local Health Units (8 in E-R and 9 in Veneto). Additional results for these variables are reported in Supplementary Table 4. Heterogeneity was measured with the tau (τ) statistic and its significance was assessed with the Cochran’s Q test. HR = subdistribution hazard ratio. CI = confidence interval. *p* = *p*-value.

**Figure 6:**
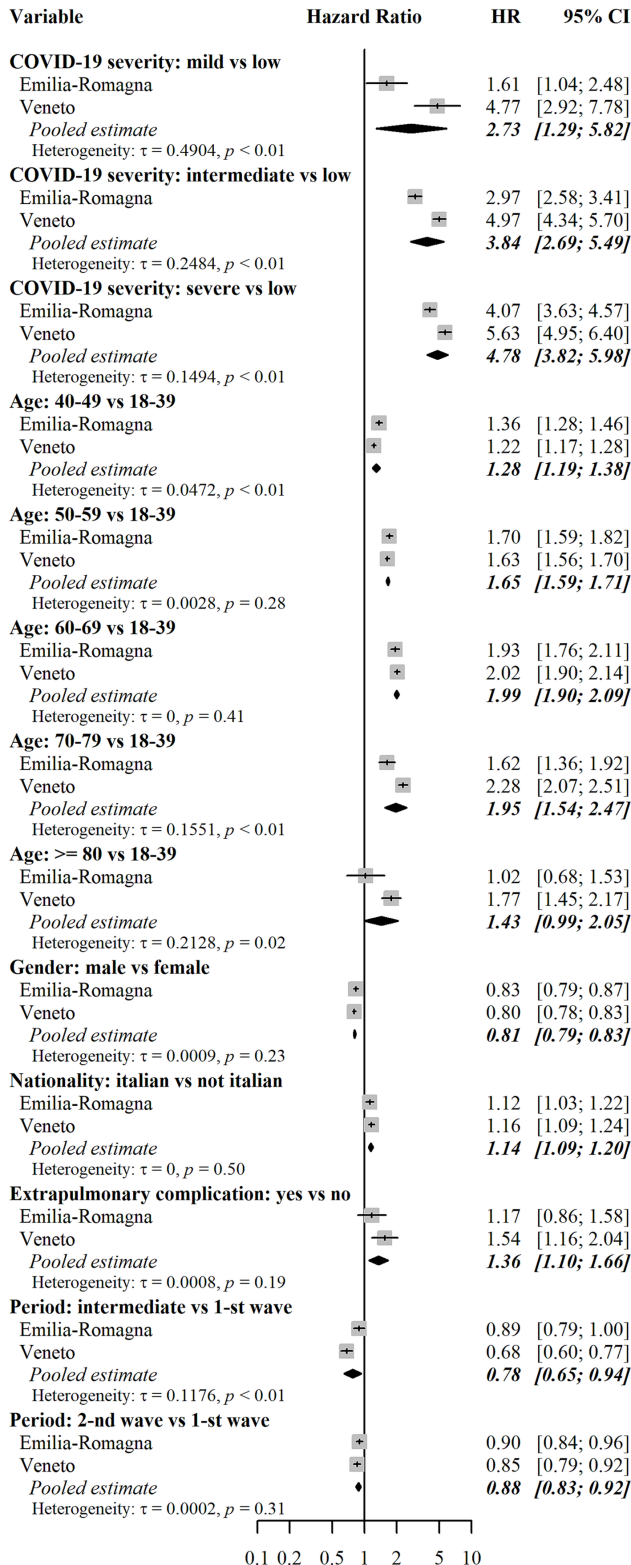
Assessment of risk factors for outpatient care. Results for each cohort were estimated using multivariable Fine-Gray subdistribution hazard models. Confidence intervals for hazard ratios in the two cohorts were calculated with the Wald method based on normal approximation and were two-sided. Pooled results were estimated using random effects meta-analysis with inverse variance weights and maximum likelihood estimator for between-study variance. The models also included the following independent variables: risk of hospitalization and death score (only in the E-R cohort), and Local Health Units (8 in E-R and 9 in Veneto). Additional results for these variables are reported in Supplementary Table 4. Heterogeneity was measured with the tau (τ) statistic and its significance was assessed with the Cochran’s *Q* test. HR = subdistribution hazard ratio. CI = confidence interval. *p* = *p*-value.

According to the confounder-adjusted pooled analysis reported in Figure 4, the major risk factor was the level of COVID-19 severity during the AP. Those with mild severity had +174% hazard of combined outcome compared to those with low severity (HR = 2.74, 95% CI = 1.49–5.02), whereas those with intermediate severity had +260% hazard (HR = 3.60, 95%CI = 2.41–5.36). Finally, those with severe COVID-19 had +321% hazard (HR = 4.21, 95%CI = 3.22–5.48). Such differences among severity groups were not homogeneous in the two regional cohorts, being HRs for Veneto higher than those for E-R. The reason for such a heterogeneity is shown in Figure 3, which describes cumulative incidence curves by severity groups in the two regional cohorts. In the E-R cohort, the risk of the combined outcome at the end of the PAP was 23.5% (95% CI = 23.0–23.9%) for low severity patients, 37.2% (95% CI = 26.6–47.4%) for mild-severity ones, 56.9% (95% CI = 52.2–61.1%) for moderate-severity ones and 66.5% (95% CI = 63.0–69.7%) for severe patients. In the Veneto cohort, the same figures were equal to 20.5% (95% CI = 20.2–20.7%), 59.5% (95% CI = 41.6–73.5%), 67.8% (95% CI = 63.6–71.7%) and 74.3% (95% CI = 70.6–77.6%), respectively. The occurrence of an acute extrapulmonary complication during the AP was associated with a higher risk (HR = 1.84, 95% CI = 1.44–2.35). Age at diagnosis was also a risk factor for the combined outcome, as the risk in higher age groups was always greater than or equal to the risk in the group of individuals aged 18–39. In particular, those aged 60–69 or 70–79 had more than a two-fold hazard (HR = 2.10, 95%CI = 2.01–2.19 and HR = 2.31, 95%CI = 1.96–2.73, respectively) and those aged ≥80 had less than a two-fold hazard (HR = 1.73, 95%CI = 1.15–2.60). Male individuals had −18% hazard of outcome compared to females (HR = 0.82, 95%CI = 0.80–0.84). Italians had a slightly higher level of risk compared to non-Italian citizens (HR = 1.09, 95%CI = 1.05–1.14). Finally, there were only minor differences between individuals diagnosed in the second epidemic wave (HR = 0.92, 95%CI = 0.88–0.97) or between individuals diagnosed in the intermediate period (HR = 0.81, 95%CI = 0.73–0.90) and those diagnosed in the first epidemic wave. The analysis of risk factors for each separate outcome (drug prescriptions or outpatient care), is reported in Figures 5, 6. Overall, the risk factors were similar to those already described for the combined outcome, with the following three exceptions. Firstly, COVID-19 severity in the AP was associated with a higher increase in the risk of outpatient care, compared to the increase in the risk of drug prescriptions. Secondly, individuals with Italian citizenship had a lower hazard of drug prescriptions (HR = 0.90, 95%CI = 0.85–0.96), whereas the opposite was observed in relation to outpatient care (HR = 1.14, 95%CI = 1.09–1.20). Thirdly, the presence of acute extrapulmonary complications during the AP was strongly associated with drug prescriptions (HR = 3.83, 95%CI = 2.96–4.95), whereas its relationship with outpatient care was of minor relevance (HR = 1.36, 95%CI = 1.10–1.66). Other minor differences in risk factor intensity were present, although they did not alter the overall interpretation of the results (Figures 5, 6).

## 4. Discussion

### 4.1. Major findings

The present study focused on the incidence and determinants of access to selected healthcare services in a largely healthy population of subjects who had a diagnosis of SARS-CoV-2 in the Emilia-Romagna and Veneto regions, Italy. The analysis covers people diagnosed in the period from February to December 2020, when vaccination against COVID-19 was not yet available. The outcome (combination of outpatient care and drug prescriptions) occurred in a high percentage of subjects (25% cumulative incidence in E-R and 21% in Veneto) during the PAP, i.e., from day 31 to day 365 after diagnosis of SARS-CoV-2. It should be however noted that the incidence in a health population in the absence of COVID-19 was not measured, leaving uncertainty about the proportion of outcomes directly attributable to the disease. Considering the two outcomes separately, outpatient care was observed more frequently than drug prescriptions (18% vs. 12% in E-R and 15% vs. 10% in Veneto). The most frequently administered drugs were cardiovascular system drugs and corticosteroids, whereas the most common category of outpatient care was the cardio-respiratory one. Hospitalization occurred more rarely (about 3%). The cumulative incidence curve grows steadily for the combined outcome and for the outpatient care outcome. For drug prescriptions, growth appears steeper in the first month of follow-up in the PAP. The curves show significant differences if the analysis is stratified by severity of acute COVID-19. In particular, subjects with severe forms showed a much steeper increase in cumulative incidence in the first part of the follow-up, especially for drug prescriptions. Risk factors associated with the combined outcome were female sex, age over 40 years, moderate to severe acute COVID-19, and acute extrapulmonary complications occurring during the AP. Although there are differences, the risk factors associated with outpatient care and drug prescriptions (considered separately) are the same as those associated with the combined outcome. Having Italian citizenship is an exception, as it was a risk factor for the prescription of drugs and a protection factor for outpatient visits in the follow-up. This finding may be related to a different use of private outpatient care. Acute COVID-19 extrapulmonary complications is another exception, as it was strongly associated with drug prescriptions, but not with outpatient care.

### 4.2. Consistency between cohorts

The results of the two cohorts, although providing quite consistent results, show a greater frequency of outcomes in E-R than in Veneto Region. The meta-analysis of the factors associated with the outcome shows heterogeneity which is high for some variables such as the severity of the acute COVID-19. In particular, the negative effect of increasing COVID-19 severity on the need for outpatient care was more intense in Veneto than in E-R. These findings can be explained by the differences in diagnostic testing policies in place in the two Regions in early 2020, when Veneto Region was the first to implement intensive diagnostic testing and contact tracing procedures, leading to a higher share of asymptomatic individuals as opposed to paucisymptomatic or weakly symptomatic ones (29). The lower incidence of outcomes observed in the Veneto cohort for subjects classified at low severity is consistent with this hypothesis. Furthermore, the cumulative incidence for mild, moderate, and severe patients was higher in Veneto than in E-R. This may depend on different long-term case management policies for hospitalized subjects in the two Regions.

### 4.3. Implications for clinical practice

The study results showed a remarkable frequency of outpatient care and drug prescriptions in the post-acute follow-up period. These data suggest that long-term effects of COVID-19 needing clinical attention occur in a substantial percentage of cases, even among a previously healthy population with low or mild severity of acute COVID-19. The latter finding may be very important for clinicians and for healthcare policy makers as, thus far, an active follow-up has often been restricted to COVID-19 patients with moderate or severe infection (3). Our data may indeed suggest the need for a greater clinical attention in the follow-up after SARS-CoV-2 infection, also in a non-hospitalized low-risk population, as other recent reports have suggested (4, 18, 23). Furthermore, in subjects with severe acute COVID-19, a high frequency of outpatient visits and prescriptions is observed in the first part of the follow-up, suggesting that there may be a continuation of the AP (defined by NICE as ongoing COVID). Our results also contribute to the evidence on risk factors for the post-COVID syndrome and are in line with previous studies carried out in other populations of COVID-19 patients (3–6, 18, 23).

### 4.4. Implications for research

Based on the results of our study, the access to outpatient care and drug prescriptions was very frequent even in a low-risk population. The significant healthcare resource consumption related to such outpatient care and administration of drugs should therefore be considered by researchers when evaluating the healthcare burden after a SARS-CoV-2 diagnosis. Moreover, our data can be useful for setting benchmarks in the levels of access to selected healthcare services, as they are referred to a large unvaccinated population at low risk of severe acute COVID-19.

### 4.5. Limitations

This study used healthcare administrative databases as data sources; therefore, only drug prescriptions and outpatient care provided by the regional healthcare systems were included in the analysis. In addition, there are no data on diagnoses of outpatient care and indications for drug prescriptions. The occurrence of selected drug prescriptions and selected outpatient care was indeed used as the outcome variable and considered as a proxy for long COVID. Therefore, the present analysis was only able to assess a variation in prescriptions and outpatient services in general terms, whereas no information on the incidence and risk factors of single drugs and outpatient services can be derived. Secondly, our results refer to unvaccinated subjects, but the epidemiological features of long COVID have changed significantly since the introduction of vaccines. Thirdly, unmeasured characteristics (e.g., environmental, lifestyle and genetic characteristics, SARS-CoV-2 variants) may have affected our results. Furthermore, outpatient care could result to some extent from the implementation of algorithms for the patient’s follow-up, regardless of persistence or recurrence of symptoms or the emergence of new ones. This limitation could have caused an overestimation of the incidence of study outcomes, particularly those related to outpatient diagnostics. Another potential limitation is in the criteria for the selection of a healthy population at low risk of severe acute COVID-19 from administrative databases. It is indeed possible that some non-healthy subjects, especially those with chronic diseases of minor clinical relevance (e.g., asthma), were included in the cohort due to the absence of relevant access to healthcare services in the one-year period before SARS-CoV-2 diagnosis. Similarly, the criteria for acute COVID-19 severity, being based on information available only for hospitalized subjects, may have been influenced by different health policies and hospital capacity during different pandemic periods. Finally, no control group of subjects who were not diagnosed with SARS-CoV-2 infection was considered, leaving uncertainty on the incidence and risk factors of outcomes directly attributable to COVID-19.

### 4.6. Strengths

The study data sources allowed for a multicenter population-based design (all cases of COVID-19 that met the inclusion criteria in two Italian Regions) and for a long follow-up period (one year from the diagnosis of SARS-CoV-2 infection). These are strengths compared to most published studies which present one or more limitations reducing the validity and generalizability of the results (3.17). The most frequent flaws of published studies refer to small or selected samples (e.g., focus on hospitalized or more symptomatic patients) and short follow-up periods (e.g., less than 12–24 weeks). Other limitations of the available studies relate to design (e.g., in surveys, people who are still unwell or who have had a long-lasting illness are more likely to participate and recall symptoms) and case definition (e.g., the use of serology as an inclusion test makes the dating of the infection inaccurate) (3.17). Moreover, the selection of low-risk subjects (i.e., no hospitalization, visit to the emergency room, prescription of specific drugs or specific outpatient visits in the year preceding the diagnosis of SARS-CoV-2 infection) makes the observed outcomes in the follow-up period largely attributable to the post-COVID syndrome.

## 5. Conclusion

Management of patients with a previous SARS-CoV-2 infection should be targeted to ongoing symptoms and new ones that have occurred. It must therefore take into account the severity of acute COVID-19 but also adapt to the clinical needs that may have emerged later on.

## Data availability statement

The datasets presented in this article are not readily available because of security measures in place to protect the privacy of participants. The data supporting the findings of this study are available at aggregated level upon reasonable request and with the written permission of Emilia-Romagna and Veneto Regions. Requests to access the datasets should be directed to EB, elena.berti@regione.emilia-romagna.it for the Emilia-Romagna cohort and to FR, francesca.russo@regione.veneto.it for the Veneto cohort.

## Ethics statement

The studies involving humans were approved by Comitato Etico Area Vasta Emilia Nord (on 8-th February 2022), Comitato Etico Area Vasta Emilia Centro (on 19-th January 2022), and Comitato Etico della Romagna (on 18-th February 2022) for the E-R cohort, and by Comitato Etico per la Sperimentazione Clinica delle Province di Verona e Rovigo (on 21-st July 2021) for the Veneto Cohort. The studies were conducted in accordance with the local legislation and institutional requirements. The Ethics Committee/Institutional Review board waived the requirement of written informed consent for participation from the participants or the participants’ legal guardians/next of kin because this study was considered an exemption to Art. 14 of the General Data Protection Regulation (GDPR), due to the disproportionate effort to provide the information to data subjects about the existence of the study processing operation and that personal (health) data were processed for scientific purposes.

## Author contributions

CG, RB, ER, ET, MM, FR, GP, SB, ES, AP, and EN: conceptualization. FB, RB, and EN: data curation. FB and EN: formal analysis. ET, MM, and FR: funding acquisition. CG, FB, RB, ER, MM, GP, SB, ES, AP, and EN: methodology. FB, ET, EB, MM, EN, MT, FR, and LC: project administration. FB and RB: software. CG, MR, ET, EB, MM, FR, and MT: supervision. FB, CG, ER, ET, EB, MM, FR, and LC: validation. FB: visualization. CG and FB: writing – original draft. CG, FB, RB, ER, MR, ET, EB, and MM: writing – review and editing. All authors contributed to the article and approved the submitted version.

## Footnotes

^1^ www.orchestra-cohort.eu

## References

[1] Lambert H Gupte J Fletcher H Hammond L Lowe N Pelling M. COVID-19 as a global challenge: towards an inclusive and sustainable future. Lancet Planet Health. (2020) 4:e312–4. doi: 10.1016/S2542-5196(20)30168-6, PMID: 32702296

[2] Adeloye D Elneima O Daines L Poinasamy K Quint JK Walker S. The long-term sequelae of COVID-19: an international consensus on research priorities for patients with pre-existing and new-onset airways disease. Lancet Respir Med. (2021) 9:1467–78. doi: 10.1016/S2213-2600(21)00286-1, PMID: 34416191 PMC8372501

[3] Nittas V Gao M West EA Ballouz T Menges D Hanson SW. Long COVID through a public health Lens: an umbrella review. Public Health Rev. (2022) 43:1604501. doi: 10.3389/phrs.2022.1604501, PMID: 35359614 PMC8963488

[4] Nguyen NN Hoang VT Dao TL Dudouet P Eldin C Gautret P. Clinical patterns of somatic symptoms in patients suffering from post-acute long COVID: a systematic review. Eur J Clin Microbiol Infect Dis. (2022) 41:515–45. doi: 10.1007/s10096-022-04417-4, PMID: 35142947 PMC8830952

[5] Stavem K Ghanima W Olsen MK Gilboe HM Einvik G. Persistent symptoms 1.5-6 months after COVID-19 in non-hospitalised subjects: a population-based cohort study. Thorax. (2021) 76:405–7. doi: 10.1136/thoraxjnl-2020-216377, PMID: 33273028 PMC7716295

[6] Davis HE McCorkell L Vogel JM Topol E. Long COVID: major findings, mechanisms and recommendations. Nat Rev Microbiol. (2023) 21:133–46. doi: 10.1038/s41579-022-00846-2, PMID: 36639608 PMC9839201

[7] Amenta EM Spallone A Rodriguez-Barradas MC El Sahly HM Atmar RL Kulkarni PA. Postacute COVID-19: an overview and approach to classification. Open forum. Infect Dis. (2020) 7:ofaa509. doi: 10.1093/ofid/ofaa509, PMID: 33403218 PMC7665635

[8] National Institute for Health and Care Excellence (NICE). COVID-19 rapid guideline: Managing the long-term effects of COVID-19 (2020). Available at: https://www.nice.org.uk/guidance/ng188 (accessed August 1, 2023). 33555768

[9] Soriano JB Murthy S Marshall JC Relan P Diaz JV. WHO clinical case definition working group on post-COVID-19 condition. A clinical case definition of post-COVID-19 condition by a Delphi consensus. Lancet Infect Dis. (2022) 22:e102–7. doi: 10.1016/S1473-3099(21)00703-9, PMID: 34951953 PMC8691845

[10] Augustin M Schommers P Stecher M Dewald F Gieselmann L Gruell H. Post-COVID syndrome in non-hospitalised patients with COVID-19: a longitudinal prospective cohort study. Lancet Reg Health Eur. (2021) 6:100122. doi: 10.1016/j.lanepe.2021.100122, PMID: 34027514 PMC8129613

[11] Ceban F Leber A Jawad MY Yu M Lui LMW Subramaniapillai M. Registered clinical trials investigating treatment of long COVID: a scoping review and recommendations for research. Infect Dis (Lond). (2022) 54:467–77. doi: 10.1080/23744235.2022.2043560, PMID: 35282780 PMC8935463

[12] Han Q Zheng B Daines L Sheikh A. Long-term sequelae of COVID-19: a systematic review and Meta-analysis of one-year follow-up studies on post-COVID symptoms. Pathogens. (2022) 11:269. doi: 10.3390/pathogens11020269, PMID: 35215212 PMC8875269

[13] Huang L Li X Gu X Zhang H Ren L Guo L. Health outcomes in people 2 years after surviving hospitalisation with COVID-19: a longitudinal cohort study. Lancet Respir Med. (2022) 10:863–76. doi: 10.1016/S2213-2600(22)00126-6, PMID: 35568052 PMC9094732

[14] Jennings G Monaghan A Xue F Mockler D Romero-Ortuño R. A systematic review of persistent symptoms and residual abnormal functioning following acute COVID-19: ongoing symptomatic phase vs. post-COVID-19 syndrome. J Clin Med. (2021) 10:5913. doi: 10.3390/jcm10245913, PMID: 34945213 PMC8708187

[15] Taquet M Geddes JR Husain M Luciano S Harrison PJ. 6-month neurological and psychiatric outcomes in 236 379 survivors of COVID-19: a retrospective cohort study using electronic health records. Lancet Psychiatry. (2021) 8:416–27. doi: 10.1016/S2215-0366(21)00084-5, PMID: 33836148 PMC8023694

[16] Xie Y Al-Aly Z. Risks and burdens of incident diabetes in long COVID: a cohort study. Lancet Diabetes Endocrinol. (2022) 10:311–21. doi: 10.1016/S2213-8587(22)00044-4, PMID: 35325624 PMC8937253

[17] Sandler CX Wyller VBB Moss-Morris R Buchwald D Crawley E Hautvast J. Long COVID and post-infective fatigue syndrome: a review. Open forum. Infect Dis. (2021) 8:ofab440. doi: 10.1093/ofid/ofab440, PMID: 34631916 PMC8496765

[18] Chen C Haupert SR Zimmermann L Shi X Fritsche LG Mukherjee B. Global prevalence of post COVID-19 condition or long COVID: a Meta-analysis and systematic review. J Infect Dis. (2022) 226:1593–607. doi: 10.1093/infdis/jiac136, PMID: 35429399 PMC9047189

[19] Angarita-Fonseca A Torres-Castro R Benavides-Cordoba V Chero S Morales-Satán M Hernández-López B. Exploring long COVID condition in Latin America: its impact on patients’ activities and associated healthcare use. Front Med. (2023) 10:1168628. doi: 10.3389/fmed.2023.1168628, PMID: 37153089 PMC10157152

[20] Louis DZ Robeson M McAna J Maio V Keith SW Liu M. Predicting risk of hospitalisation or death: a retrospective population-based analysis. BMJ Open. (2014) 4:e005223. doi: 10.1136/bmjopen-2014-005223, PMID: 25231488 PMC4166245

[21] Stevens GA Alkema L Black RE Boerma JT Collins GS Ezzati M. Guidelines for accurate and transparent health estimates reporting: the GATHER statement. Lancet. (2016) 388:e19–23. doi: 10.1016/S0140-6736(16)30388-9, PMID: 27371184

[22] Nobili A D’Avanzo B Tettamanti M Galbussera AA Remuzzi G Fortino I. Post-COVID condition: dispensation of drugs and diagnostic tests as proxies of healthcare impact. Intern Emerg Med. (2023) 18:801–9. doi: 10.1007/s11739-023-03228-5, PMID: 36944811 PMC10030070

[23] Al-Aly Z Xie Y Bowe B. High-dimensional characterization of post-acute sequelae of COVID-19. Nature. (2021) 594:259–64. doi: 10.1038/s41586-021-03553-9, PMID: 33887749

[24] Pfaff ER Madlock-Brown C Baratta JM Bathia A Davis H Girvin A. Coding long COVID: characterizing a new disease through an ICD-10 lens. BMC Med. (2023) 21:58. doi: 10.1186/s12916-023-02737-6, PMID: 36793086 PMC9931566

[25] Kalbfleisch JD Prentice RL. Competing risks and multistate models In: The statistical analysis of failure time data. Hoboken, NJ: J. Wiley (2002). 247–77.

[26] Aalen O. Nonparametric estimation of partial transition probabilities in multiple decrement models. Ann Stat. (1978) 6:534–45. doi: 10.1214/aos/1176344198

[27] Fine JP Gray RJ. A proportional hazards model for the subdistribution of a competing risk. J Am Stat Assoc. (1999) 94:496–509. doi: 10.1080/01621459.1999.10474144

[28] Deeks JJ Altman DG Bradburn MJ. Statistical methods for examining heterogeneity and combining results from several studies in meta-analysis In: Egger M Davey Smith G Altman DG, editors. Systematic reviews in health care: Meta-analysis in context. London (UK): BMJ Publication Group (2001). 285–312.

[29] Mugnai G Bilato C. Covid-19 in Italy: lesson from the Veneto region. Eur J Intern Med. (2020) 77:161–2. doi: 10.1016/j.ejim.2020.05.039, PMID: 32527610 PMC7253944

