# Supplementary material for: The incidence and risk factors of selected drug prescriptions and outpatient care after SARS-CoV-2 infection in low-risk subjects: a multicenter population-based cohort study
Source: Front Public Health. 2023 Oct 4;11:1241401. doi: 10.3389/fpubh.2023.1241401 (PMC10582710; doi:10.3389/fpubh.2023.1241401)
Supplement: Supplementary file 1 [file Table_1.docx]

## **Supplementary Table 1. Criteria for the identification of selected drug prescriptions and selected outpatient care**

| **Selected drug prescriptions** | **Anatomical Chemical Therapeutical (ATC) codes (descriptions)** |
| --- | --- |
| Cardiovascular system / antithrombotic | B01 (antithrombotic agents); C01A (cardiac glycosides); C01B (antiarrhythmics, class I and III); C02 (antihypertensives); C03 (diuretics); C07 (beta blocking agents); C08 (calcium channel blockers); C09 (agents acting on the renin-angiotensin system) |
| Antidiabetic | A10 (drugs used in diabetes) |
| Nervous system | N06A (antidepressants); N06DA (anticholinesterases) |
| Respiratory system | R03A (adrenergics, inhalants); R03B (other drugs for obstructive airway diseases, inhalants) |
| Oxygen | V03AN01 (oxygen) |
| Corticosteroids | H02 (corticosteroids for systemic use) |
| **Selected outpatient care** | **Regional outpatient codes (descriptions)** * |
| Cardio-respiratory | 87.41, 87.41.1, 87.44.1, 88.73.3, 88.92, 88.92.1 (chest imaging); 88.72.1, 88.72.2, 88.72.3 (cardiac ultrasound imaging); 89.01.3, 89.7A.3 (cardiological ambulatory visits); 89.01.L, 89.7B.9 (pneumological ambulatory visits); 89.37.1, 89.37.2, 89.37.3, 89.38.2, 89.38.3, 89.38.5, 89.44.1, 89.44.2, 89.65.1, 89.65.2, 89.65.3, 89.65.4, 89.65.5, 89.65.6, 89.66 (pneumological diagnostics); 89.50, 89.52, 89.54 (electrocardiography); 93.95^a^, 93.95.1 & 93.95.2^b^ (oxygen); 93.18.1, 93.18.2, 93.36 (respiratory and cardiological rehabilitation) |
| Vascular | 88.73.5, 88.77.1, 88.77.2, 88.77.3, 88.77.4^b^, 88.77.5^b^, 88.77.6 ^b^, 88.77.7^b^ (peripheral vascular ultrasound imaging); 89.7A.2 (angiological ambulatory visits) |
| Neuro-psychiatric | 89.01.C, 89.13 (neurological ambulatory visits); 93.89.2, 93.89.3 (training for cognitive disorders); 89.01.M^b^, 94.12.1^a^, 94.19.1, 94.09 (psychiatric ambulatory visits) |
| Rehabilitation-motor | 938901^a^, 89.01.W, 89.7B.2, 89.94.2^b^, 89.94.3^b^ (rehabilitation-motor ambulatory visits) |
| Nephrology | 39.95.1, 39.95.2, 39.95.3, 39.95.4, 39.95.5, 39.95.6, 39.95.7, 39.95.8, 39.95.9 (hemodialysis), 89.01.B, 89.7B.4 (nephrological ambulatory visits); 92.03.3 (renal imaging) |
| Diabetes | 890180^a^, 89.01.8^b^, 897A80^a^, 89.7A.8^b^ (diabetic ambulatory visits); 90.28.1 (glycated hemoglobin) |

Notes: ATC = Anatomical Chemical Therapeutical classification of the World Health Organization, which can be accessed at the following link: https://www.whocc.no/atc (last accessed on 26th May 2023). * Regional outpatient codes of the Emilia-Romagna Region are described in the Nomenclatore Tariffario Regionale della Specialistica, which can be accessed at the following link: https://salute.regione.emilia-romagna.it/ssr/strumenti-e-informazioni/nomenclatore-tariffario-rer (last accessed on 26th May 2023). Regional outpatient codes of the Veneto Region are described in the Nomenclatore Tariffario Prestazioni Specialistiche Ambulatoriali, which can be accessed at the following link: https://salute.regione.veneto.it/web/fser/catalogo-veneto-prescrivibile#:~:text=Il%20Catalogo%20Veneto%20del%20Prescrivibile,all'utilizzo%20della%20ricetta%20dematerializzata (last accessed on 26th May 2023). ^a^ = this outpatient code exists only in Emilia-Romagna Region’s databases; ^b^ = this outpatient code exists only in Veneto Region’s databases.
