# Supplementary material for: The incidence and risk factors of selected drug prescriptions and outpatient care after SARS-CoV-2 infection in low-risk subjects: a multicenter population-based cohort study
Source: Front Public Health. 2023 Oct 4;11:1241401. doi: 10.3389/fpubh.2023.1241401 (PMC10582710; doi:10.3389/fpubh.2023.1241401)
Supplement: Supplementary file 2 [file Table_2.docx]

## **Supplementary Table 2. Data extraction criteria for COVID-19 severity, acute extrapulmonary complications and other drug prescriptions**

| **Respiratory diagnoses and ventilation procedures**  **(acute phase)** | **International Classification of Diseases (ICD-9-CM) codes / Diagnosis Related Groups (DRG) / other criteria** |
| --- | --- |
| Acute respiratory insufficiency | ICD-9-CM principal and secondary diagnosis codes in hospital records: 518.81-82, 518.8, 518.9 (acute respiratory insufficiency) * |
| Pneumonia or acute lower respiratory tract infections | ICD-9-CM principal and secondary diagnosis codes in hospital records: 464.1-2, 466, 490, 491.22, 519.7 (acute tracheitis, bronchitis and bronchiolitis); 480-486, 487.0 (pneumonia); 487.1 (influenza with respiratory manifestations) * |
| Other respiratory disease | ICD-9-CM principal and secondary diagnosis codes in hospital records: 460-519, except 464.1-2, 466, 480-486, 487.0, 487.1, 490, 491.22, 518.81-82, 518.84, 518.9, 519.7 * |
| Intensive care unit stay | At least one day in intensive care units (national unit code 049) |
| Sub-intensive care unit stay | At least one day in sub-intensive care units (national unit code 094) |
| Oxygen therapy | ICD-9-CM principal and secondary diagnosis codes in hospital records: V46.2 OR  ICD-9-CM procedure codes in hospital records: 93.91, 93.96, 93.99 |
| Non-invasive ventilation | ICD-9-CM procedure codes in hospital records: 93.90 |
| Invasive ventilation | ICD-9-CM procedure codes in hospital records: 96.70, 96.71, 96.72 |
| Viral illness DRGs | Hospital records classified in DRGs 421 or 422 (CMS-DRG version 24.0) |
| **Acute extrapulmonary complications**  **(acute phase)** | **International Classification of Diseases (ICD-9-CM) principal and secondary diagnosis codes** |
| Vascular-haemorrhagic-thrombotic complications | 286.6 (defibrination syndrome); 415 (acute pulmonary heart disease); 430-436, 437.1, 437.6-9 (cerebrovascular disease), 444, 451-453 (arterial or venous embolism and thrombosis) |
| Cardiac complications | 410 (acute myocardial infarction); 411 (acute and subacute forms of ischemic heart disease); 422 (acute myocarditis) |
| Neurological complications | 357.0 (acute polyneuritis); 357.82, 359.81 (critical illness polyneuropathy or myopathy) |
| Septicemia | 038 (septicemia), 785.52 (septic shock), 995.90-92 (systemic inflammatory response syndrome and sepsis) |
| Acute organ failure complications | 584, 586 (acute renal failure); 348.3 (encephalopathy), 428.0-1, 428.20-21, 428.23, 428.30-31, 428.33, 428.40-41, 428.43, 428.9 (acute heart failure); 570 (acute and subacute necrosis of liver) |
| **Other drug prescriptions** | **Anatomical Chemical Therapeutical (ATC) codes** |
| Antibacterial | J01 (antibacterials for systemic use) |
| Hydroxychloroquine | P01BA02 (hydroxychloroquine) |

Notes: ICD-9-CM = International Classification of Diseases, 9th revision - Clinical Modification, Versione Italiana 2007, which can be accessed at the following link: https://www.salute.gov.it/imgs/C_17_pubblicazioni_2251_allegato.pdf (last accessed on 26th May 2023). * = Diagnosis codes 480.4, 518.9, and 519.7 were introduced by the Italian Ministry of Health in 2021. DRG = Diagnosis Related Group. ATC = Anatomical Chemical Therapeutical classification of the World Health Organization, which can be accessed at the following link: https://www.whocc.no/atc (last accessed on 26th May 2023).
