# Supplementary material for: The incidence and risk factors of selected drug prescriptions and outpatient care after SARS-CoV-2 infection in low-risk subjects: a multicenter population-based cohort study
Source: Front Public Health. 2023 Oct 4;11:1241401. doi: 10.3389/fpubh.2023.1241401 (PMC10582710; doi:10.3389/fpubh.2023.1241401)
Supplement: Supplementary file 3 [file Table_3.docx]

## **Supplementary Table 3. Monthly cumulative incidence of outcomes**

| Months in the  Post-Acute Phase | **Combined outcome^1^** | | **Drug prescriptions** | | **Outpatient care** | |
| --- | --- | --- | --- | --- | --- | --- |
|  | **Emilia-Romagna** | **Veneto** | **Emilia-Romagna** | **Veneto** | **Emilia-Romagna** | **Veneto** |
| 1 | 4.4% (4.2-4.7%) | 3.8% (3.7-3.9%) | 2.2% (2.1-2.4%) | 1.5% (1.5-1.6%) | 2.6% (2.5-2.8%) | 2.6% (2.5-2.7%) |
| 2 | 7.4% (7.2-7.7%) | 6.4% (6.2-6.6%) | 3.4% (3.2-3.6%) | 2.5% (2.4-2.6%) | 5.0% (4.8-5.2%) | 4.6% (4.5-4.7%) |
| 3 | 10.3% (9.9-10.6%) | 8.7% (8.5-8.9%) | 4.6% (4.4-4.9%) | 3.5% (3.4-3.6%) | 7.1% (6.8-7.4%) | 6.4% (6.2-6.5%) |
| 4 | 12.8% (12.4-13.1%) | 10.6% (10.4-10.8%) | 5.7% (5.5-5.9%) | 4.3% (4.1-4.4%) | 9.0% (8.7-9.3%) | 7.8% (7.6-7.9%) |
| 5 | 15.0% (14.7-15.4%) | 12.3% (12.1-12.5%) | 6.8% (6.5-7.0%) | 5.1% (4.9-5.2%) | 10.7% (10.4-11.0%) | 9.0% (8.8-9.2%) |
| 6 | 17.1% (16.7-17.5%) | 14.0% (13.7-14.2%) | 7.6% (7.3-7.9%) | 5.8% (5.7-6.0%) | 12.3% (11.9-12.6%) | 10.2% (10.0-10.4%) |
| 7 | 18.8% (18.4-19.3%) | 15.4% (15.2-15.6%) | 8.5% (8.2-8.8%) | 6.5% (6.3-6.7%) | 13.5% (13.2-13.9%) | 11.3% (11.0-11.5%) |
| 8 | 20.4% (19.9-20.8%) | 16.7% (16.5-16.9%) | 9.3% (9.0-9.6%) | 7.1% (7.0-7.3%) | 14.6% (14.3-15.0%) | 12.2% (12.0-12.4%) |
| 9 | 21.7% (21.3-22.2%) | 18.1% (17.8-18.3%) | 10.0% (9.7-10.3%) | 7.8% (7.6-8.0%) | 15.6% (15.2-16.0%) | 13.2% (13.0-13.4%) |
| 10 | 23.3% (22.9-23.8%) | 19.6% (19.3-19.9%) | 10.9% (10.6-11.2%) | 8.6% (8.4-8.8%) | 16.7% (16.4-17.1%) | 14.2% (14.0-14.5%) |
| 11 | 24.9% (24.5-25.4%) | 21.2% (20.9-21.5%) | 11.9% (11.5-12.2%) | 9.5% (9.3-9.7%) | 17.9% (17.5-18.3%) | 15.4% (15.1-15.6%) |

Notes: monthly estimates of cumulative incidence function curves with 95% confidence interval (CI) are reported for the two cohorts. ^1^ = the combined outcome includes selected drug prescriptions and selected outpatient care, whichever came first. Confidence intervals were calculated with the asymptotic Aalen method.
